# Health Care and Social Work Students’ Experiences With a Virtual Reality Simulation Learning Activity: Qualitative Study

**DOI:** 10.2196/49372

**Published:** 2023-09-20

**Authors:** Nikolina Helle, Miriam Dubland Vikman, Tone Dahl-Michelsen, Silje Stangeland Lie

**Affiliations:** 1 Institute of Health Faculty of Health Sciences VID Specialized University Stavanger Norway; 2 Institute of Health Faculty of Health Sciences VID Specialized University Bergen Norway

**Keywords:** virtual reality, virtual reality simulation, learning, experiences, health care and social work, higher education, health care, social work

## Abstract

**Background:**

Virtual reality is used to an increasing extent in various fields and is now making inroads into health and social education. Virtual reality simulation can provide a safe and controlled environment for students to practice and master skills that are transferable to real-world situations without putting patients, clients, or themselves at risk of any harm. Virtual reality simulation using 360° videos represents a novel approach to simulation in health care and social work education, and this inspired our interest in exploring students’ experiences with such a learning activity.

**Objective:**

The aim of this study was to explore occupational therapy, social education, nursing, and social work students’ experiences with virtual reality simulation as a learning activity in an interdisciplinary subject.

**Methods:**

The data were collected through 6 semistructured focus groups with 28 students. We conducted the focus groups after the students from the 4 education programs had participated in the virtual reality simulation at 3 campuses at a specialized university in Norway. Each focus group interview was facilitated by 1 moderator and 1 facilitator, a combination of experienced researchers and novices. We followed a qualitative design using the 6-step thematic analysis described by Braun and Clarke.

**Results:**

The analysis revealed 3 overall themes for students’ experiences with the virtual reality simulation. The first theme, *360° videos provide observations for individual learning*, illustrates how learning can take place through the students’ experiences with sensory inputs and observations from the 360° videos. Students experienced that the video enabled them to individually reflect and achieve learning from what was considered a clinically relevant video. The second theme, *360° videos activate emotional learning*, demonstrates how the students experienced emotional engagement when watching the 360° videos. The degree of realism provided in the video was considered as important for the students’ learning. The last theme, *Debrief sessions enhance comprehensive learning*, pinpoints how the students experienced learning through reflective discussions with other students after watching the 360° videos. Students claimed this process to be a vital part of the learning activity.

**Conclusions:**

Virtual reality simulation represents a promising learning activity to enhance the professional learning of health care and social work students. It offers opportunities for individualized learning through observations, and it also engages students emotionally in the learning process. The combination of 360° videos and group discussions in virtual reality appears promising to enhance professional learning outcomes and competence, which may contribute to improved health care and social work services.

## Introduction

### Background

Immersive 360° videos watched using virtual reality headsets are generally accessible and represent a financially feasible alternative to other types of virtual reality for use in health and social care education [1]. Because virtual reality headsets make it possible to block out the surrounding physical world, they increase students’ sense of immersion and their feeling of being present in the scenario portrayed [2]. When using 360° videos for virtual reality simulation, students can experience different scenarios as observers. This can provide them with authentic experiences that may increase their engagement with learning by enabling them to consider new perspectives and may promote creativity in the learning process [3]. Observation is an important skill that can be cultivated and refined through training and deliberate practice [4]. As future health care and social work professionals, students will encounter a variety of complex situations in which observation is essential. By training their observational skills, they can identify signs of health issues or challenges for patients, clients, or users. This enables them to provide appropriate support or treatment tailored to individual needs. The experiences from a previous study [5] demonstrate that the requirements for increased competence and training in new observational skills can be met by providing a combination of theory and simulation. Clinical observations rely on the health care workers’ assessment and analysis. A broad spectrum of knowledge and observational skill is necessary to recognize signs and symptoms of a deteriorating patient condition [5].

Thus, virtual reality simulation can provide a safe and controlled environment for individuals to practice and master skills that are transferable to real-world situations. It can allow health care and social work students to experience challenging situations without putting clients, patients, or themselves at risk of harm [6]. Virtual reality simulation, starting with watching 360° videos using virtual reality headsets, is a novel approach to simulation in health and social care education, and this inspired our interest in exploring students’ experiences with this approach as a learning activity.

We developed and conducted virtual reality simulation as a learning activity in an interdisciplinary setting for the bachelor programs of nursing, occupational therapy, social education, and social work. The development of the 360° videos and corresponding learning activities was reported in an earlier article from the project [7]. The 360° videos and procedure followed for the virtual reality simulation can be accessed on the web page for “Solstien 3” [8]. The aim of using virtual reality simulation was to develop the students’ soft skill competencies. As this type of virtual reality simulation is novel in health and social care educational programs, it is of great value to explore students’ experiences with this learning activity.

Watching immersive 360° videos in virtual reality headsets as the beginning of a learning activity may appeal to a broad range of learners. Therefore, it has the potential to be an effective learning tool in health care and social work education [1]. Formosa et al [9] studied virtual reality–based simulation in psychology education and found it promising. Nevertheless, the application and perceived benefits of virtual reality simulation may be significantly hindered depending on the students’ age and their overall perception of virtual reality as a teaching method [9]. Another study found that virtual reality simulation is engaging and may be effective for nursing students to learn clinical reasoning [10]. In an earlier study, Lie et al [7] explored how virtual reality can stimulate emotions and, thereby, facilitate learning in higher health care education from the faculty’s point of view. The authors indicated that faculty members emphasize that virtual reality simulation needs to be contextualized in educational programs. Further, they find that allowing students to reflect in a safe setting with faculty members is vital [7]. Blair et al [1] suggested that future research should explore the application of pedagogical theory with immersive 360° video experiences and that further interdisciplinary studies exploring the acceptability and effective utilization of this technology would be of value. Thus, it is vital to explore students’ experiences with watching 360° videos using virtual reality headsets, which is followed by group debrief sessions, as a learning activity in an interdisciplinary subject.

The majority of the research on virtual reality simulation in health education has focused on technical skills, such as surgical skills [11]. Although virtual reality is increasingly used in simulation-based training for clinical skills and role-playing in medical education [12], there remains a research gap with regard to its use for teaching soft skills, such as ethical reflection and communication, in higher education for health care and social work [13,14]. Increased knowledge of students' experience with this kind of pedagogical approach may be of importance for further development and use of simulation-based training in soft skills. It can also contribute to identifying what students consider useful learning outcomes in a simulation-based learning activity. This can be of value for health care and social work education in developing simulation-based learning activities that students consider as relevant for their future practice.

### Theoretical Background for Using Virtual Reality Simulation as a Learning Activity

Virtual reality simulation can facilitate training to acquire soft skill competence, which is a crucial requirement for all health care and social work students and practitioners. Training can be defined as a “systematic effort to impart knowledge, skills, attitudes (...) with the end goal of improved performance. To achieve this broad goal, training must lead to change in some (or all) of the following characteristics of the trainee: knowledge, patterns of cognition, attitudes, motivation, and abilities” [15]. Illeris’ comprehensive theory of learning emphasizes the importance of both the individual and societal context in which learning takes place. According to Illeris, learning is a complex and dynamic process that involves interaction between the individual, environment, and activity being undertaken. He identified the following 3 dimensions of learning: cognitive, emotional, and social [16,17].

The *cognitive dimension* refers to the acquisition of knowledge and understanding through thinking, reasoning, and problem solving, while the *emotional dimension* refers to the affective and emotional aspects of learning, such as motivation, feelings, and attitudes. Finally, the *social dimension* refers to the social and cultural factors that influence learning, including relationships, communication, and group dynamics. All 3 dimensions of learning are interconnected and influence each other [18]. The internal acquisition process implies that there is content that must be learned and that there is motivation to learn. The content is part of the acquisition process because the learner must have something to learn, while motivation refers to the mobilization of mental energy to acquire new knowledge and is also a part of the acquisition process. The interaction dimension of learning deals with communication, cooperation, and society. Further, the interaction process is about the relationship between the learner and the outside world. The learner receives various impulses through their senses. The internal acquisition process involves the recording of the impulses from the interaction process in a mental schema (something that has been developed through previous learning), meaning that it builds on previous learning. The overall learning that can be acquired in a learning situation depends on functionality (content), sensitivity (driving force), and interaction [18].

Thus, learning is about more than just the content to be learned: It is shaped by the interaction that occurs in the learning situation and the motivation of the individual [19]. If students perceive the interaction that occurs during a learning situation (ie, the group debrief session after watching a 360° video) as a positive and motivational experience, it will affect their learning positively. In contrast, a negative interaction experience and lack of motivation can affect students’ learning negatively and make them less likely to remember, use, or build on the content. Illeris’ theory of learning emphasizes the complexity and dynamic nature of the learning process and the importance of the individual and context in which learning takes place. It offers a holistic and comprehensive approach to understanding how individuals learn and how to support their learning [20].

### Aim

The aim of this study was to explore occupational therapy, social education, nursing, and social work students’ experiences with a virtual reality simulation as a learning activity in an interdisciplinary subject.

## Methods

### Design

We used a qualitative design and collected data by means of semistructured focus groups with students who participated in the learning activity.

### Context

#### Description of the “Solstien 3” Virtual Reality Simulation as a Learning Activity

We developed 360° videos to be watched in virtual reality headsets as a part of a larger project [8]. The VR simulation conformed to the following procedure: provision of information, short brief viewing of the 360° video, and student group debrief discussions led by a facilitator. The group debrief discussions followed a guide for the simulation setup and focused on identifying and reflecting on ethical dilemmas and on one’s own values and philosophy of life as well as on the significance these aspects have for one’s professional learning. We based the design of the debrief discussion guide on the Promoting Excellence and Reflective Learning in Simulation (PEARLS) simulation guide. PEARLS is an integrated conceptual framework that is used to structure health care simulation debriefs [21]. Importantly, the virtual reality simulation used in this study is a learning activity that consists of the following 2 main components: the 360° videos and the subsequent group debrief discussion.

The learning activity was conducted for all 4 selected education programs as part of an interdisciplinary subject called “Philosophy of life, values, and relationships in professional practice” on 4 separate days in May 2022. The activity correlated with the syllabus of the subject. However, as this was a pilot study, the activity was conducted in addition to the regular curriculum, and, therefore, student participation was voluntary.

#### Description of the Scenario

In the 360° video watched as the starting point of the virtual reality simulation, a service provider visits a refugee family to follow up on the father’s mental health. However, during the visit with the family, the father bursts out in anger directed toward the service provider. His anger relates to a letter he received from the kindergarten his child attends. The video was filmed based on a carefully thought-out screenplay developed by an interdisciplinary team during the earlier stages of the aforementioned larger project. It included professional actors playing the family members and an actual health care provider playing the health care provider as an amateur actor. The 360° camera used to film the video was placed in the middle of the scene such that the student or watcher could experience the situation as an observer.

### Recruitment

All students in the first year of their bachelor studies were invited to participate in the pilot during spring 2021. The approximate numbers of students invited were 250 nursing, 40 occupational therapy, 90 social education, and 60 social work students. The pilot was conducted as an addition to the regular curriculum in a subject all study programs conducted in the second semester. A total of 35 students from the 4 selected undergraduate programs (nursing, occupational therapy, social education, and social work) voluntarily participated in the pilot “Solstien 3” virtual reality simulation, and we recruited participants for the focus groups from this set of students. The groups consisted of students from the various undergraduate programs. All students were asked to participate in the focus groups before they took part in the learning activity; they were given all the relevant information, and those who agreed signed a consent form immediately after.

### Data Collection

The focus group guide was developed based on the objectives of the larger project, and one important goal of the project was student participation in all phases of the project. For this reason, the topics for discussion were formulated in an open manner, and the discussion focused on the students’ experiences, their recommendations, and other matters raised by the participants, as it may be of great value to explore one’s own field of work to improve the quality [22,23]. Focus groups as a method for data collection are characterized by a nondirective style of interviewing that encourages discussion and the expression of a variety of viewpoints. It involves open-ended questions to foster conversation and diverse perspectives [24]. The students were encouraged to speak freely, and the facilitator encouraged topical discussions among the participants in each focus group. The facilitators addressed the following topics: students’ experiences with the 360° video in the virtual reality headsets, their experiences with the virtual reality simulation in full, how they felt that VR simulation contributes to achieving learning outcomes, and what advice they had to improve the virtual reality simulation learning activity. During the focus groups, no confidential information was addressed (such as health issues and other personal matters).

In total, 6 focus groups were conducted with a total of 28 students (22 women and 6 men; 20-29 years old). The interviews lasted for 45 minutes to 90 minutes and were audio-recorded. We conducted the focus groups after the students had participated in the virtual reality simulation at 3 campuses of a Norwegian specialized university. The focus groups were conducted directly after the pilot. The group composition was affected by the 7 students who chose not to participate in the focus groups. Therefore, the groups were composed based on practical reasons and not systematically divided into single or interdisciplinary groups. This led to 4 of the groups involving students from 1 education program, and 2 of the groups were of an interdisciplinary mix; see Table 1. Each focus group was facilitated by 1 moderator and 1 facilitator, a combination of experienced researchers and novices. Authors NH, MDV, and SSL were moderators, accompanied by project participants mentioned in the Acknowledgements section. Each moderator used the same semistructured interview guide, thus ensuring consistency in the data collection process. The interview guide is presented in Multimedia Appendix 1.

**Table 1:** Overview of the focus groups and participants.

Focus group characteristics				Students, n
**#1 (n=7)**				
	**Education program**			
		Social education	7	
	**Gender**			
		Female	5	
		Male	2	
**#2 (n=3)**				
	**Education program**			
		Nursing	3	
	**Gender**			
		Female	3	
		Male	0	
**#3 (n=6)**				
	**Education program**			
		Social work	3	
		Nursing	3	
	**Gender**			
		Female	4	
		Male	2	
**#4 (n=4)**				
	**Education program**			
		Occupational therapy	3	
		Social education	1	
	**Gender**			
		Female	3	
		Male	1	
**#5 (n=3)**				
	**Education program**			
		Occupational therapy	3	
	**Gender**			
		Female	3	
		Male	0	
**#6 (n=5)**				
	**Education program**			
		Occupational therapy	5	
	**Gender**			
		Female	4	
		Male	1	

### Data Analysis

In our study, we used thematic analysis to identify themes within our data set (transcribed focus groups). Thematic analysis is a method that involves identifying, analyzing, and reporting patterns within a set of data [23]. This methodology is flexible when it comes to using theories during the analysis process [25]. The goal of our thematic analysis was to remain close to the data and to abstract themes without being constrained by theoretical assumptions during the initial analysis phases. We followed the steps of thematic analysis described by Braun and Clarke [25].

In the first phase, all authors read through the transcribed focus groups and highlighted the words and phrases related to the students’ experience with the virtual reality simulation. Second, we coded the identified meaning units. Third, we categorized all the identified codes to classify differences and similarities in the text. Fourth, we reviewed all the transcripts to look for examples of how each of the themes or categories manifested in the text. The examples were assigned to the appropriate themes to highlight attributes within each specific theme and identify patterns in the data with respect to the students’ experiences with the virtual reality simulation as different learning experiences. Fifth, the obtained categories and themes were presented during analysis seminars with all authors and discussed until consensus was reached. Sixth, we began the completion and final review of the report.

Trustworthiness refers to the extent to which the results of a qualitative study can be considered reliable and credible. In this study, trustworthiness was established through the following strategies. First, we included the full research team in the analysis process. The tentative codes, categories, and themes were discussed during several analysis seminars and were revised multiple times until consensus was reached. Second, we have provided detailed and thorough descriptions of the study setting, students, and data collection and analysis processes to ensure transparency in our analysis. We followed the Consolidated Criteria for Reporting Qualitative Studies (COREQ) 32-item checklist [26]; see Multimedia Appendix 2 and Multimedia Appendix 3.

### Ethical Considerations

This project is registered with the Norwegian Agency for Shared Services in Education and Research (Sikt; ref. 423788), and all the data were collected and stored according to their guidelines. All the students who participated in the pilot virtual reality simulation were asked to participate in the focus groups. Those who agreed signed informed consent forms (28 of 35). They were informed of their ability to withdraw from the study at any time without any negative consequences. The data were anonymized and stored securely in line with the requirements of Sikt. No compensation was provided to the students.

## Results

### Themes

The analysis resulted in the identification of 3 overall themes for the students’ experiences with learning through the virtual reality simulation. The first theme relates to how the sensory inputs in the 360° videos provided the students with observations to facilitate their individual learning. The second theme concerns the students’ experiences with emotional activation during the virtual reality simulation. Finally, the last theme relates to how the students experienced comprehensive learning through the discussion conducted after they had watched the 360° video. According to the students, the virtual reality simulation influenced their learning process through both the 360° video and the group debrief sessions. The results are described in more detail in the following subsections. For an overview of the themes and content, see Table 2.

**Table 2:** Overview of themes.

Overall themes	Codes
360° videos provide observations for individual learning	Visual learning Sensory learning Observation Experience Disturbance Lack of realism
360° videos activate emotional learning	Overwhelming Engagement Emotional experience Realistic Empathy Intuition Safety
Debrief sessions enhance comprehensive learning	Discussion Different perspectives Facilitation Safe learning space Self-consciousness Critical thinking

### 360° Videos Provide Observations for Individual Learning

The students highlighted the observational experiences as an important factor for their learning in the “Solstien 3” virtual reality simulation. The context of the video was perceived as relevant, and the visual and auditive inputs provided by the 360° video through the virtual reality headset afforded the students several aspects of the situation upon which to reflect. Watching the 360° video on a virtual reality headset was highlighted as exciting by the students, and it led to experiences and reactions they considered important for further reflection. Students reported having a sense of being present in the scenario in the video. The virtual reality simulation provided immersive visual and auditive inputs, which served as a starting point for interpreting the observed body language of the individuals in the video. In addition, the students reflected on the communication strategies they identified in the video as well as other observations about the actors in relation to the video scenario. Their experiences are illustrated by the following quotation:

You get to watch how people are reacting, you observe their body language, and you get an illusion of being in the room in a completely different way compared to watching a scene on a flat-screen TV.

Student 3, Interview 5

The visual and auditive impressions from the 360° video facilitated students’ observations as part of their individual reflection and learning. They were able to make observations regarding the surroundings and appearances using the virtual reality headset rather than only focusing on the actions of the actors in the video. They observed the client’s apartment (ie, whether it was tidy, what kind of lifestyle the furniture connoted, and the general appearance [lifestyle] of the characters). These observations were included in the students’ overall assessment of the situation, which is reflected by a student who highlighted that “scanning“ the room for these details informed them of the situation. These sensory inputs were useful for their learning experience. Several students pointed out that they observed and reflected when using virtual reality in a different way than they usually did in traditional written case assignments.

Although all the students had watched the exact same video and course of action, they still had different experiences, reflections, and points of focus. For example, one of the students was irritated by the service provider’s behavior in the video, which led her to reflect upon her own reaction. The student highlighted this learning experience as important by challenging her own feelings and attitudes toward her future work. Other students, however, were not irritated but impressed by the professional behavior of the service provider, reflecting how students had individual and varying learning experiences.

Being a “fly on the wall” was an expression used by several students when describing how they learned through observations from the 360° videos. The observer role came naturally to them while watching the actors’ reactions and actions, and they were not able to influence the portrayed situation. This more passive role of an observer created distractions for some students, which were typically expressed as “I was on the outside and looked into the situation.” However, in contrast, several students reported that they could focus more on the interaction and reactions of the actors when they themselves did not play an active role in the situation. This more “passive” role forced them to observe and reflect upon what was going on in the video. Other students described having an experience of being personally involved during the events in the portrayed video situation, which was described as a unique experience:

You get the feeling that you are there, in the situation. Now, I'm standing next to someone being yelled at by a very angry man. (...) What will happen next? What will be her (the service provider’s) next move?

Student 3, Interview 6

Most of the students stated that the situation portrayed felt realistic. However, not everyone agreed. Some were disturbed by external factors, such as the presence of the other students and noise, which hindered their experience of the realism of the portrayed scenario.

### 360° Videos Activate Emotional Learning

Students were emotionally activated during the virtual reality simulation, and emotions such as fear, empathy, sympathy, stress, discomfort, and irritation were experienced as responses to the situation portrayed in the 360° video. The whole process, which includes both the virtual reality observations through the headsets and the subsequent group debrief discussion, was perceived to be engaging.

The 360° video as the starting point of the whole virtual reality simulation learning activity was considered engaging. The realism enabled by the actors and portrayed scenario was highlighted as an important factor that created emotional engagement. The students reacted with surprise and discomfort when, for instance, one of the actors was aggressive. His aggression and angry screaming made many of the students feel the need to physically withdraw. They were also somewhat surprised by his behavior and were “alarmed” when he looked directly at the camera and approached them as an observer. The element of surprise afforded by the unexpected course of action activated their emotional reactions:

I wanted to withdraw a bit. I felt like, ‘Okay, it's completely fine that you're frustrated and show a bit of aggression and irritation, but can we keep a little bit of distance between us?’

Student 4, Interview 4

In contrast, one of the students stated that the fact that the facilitator provided information about the scenario in advance created distance from the same and reduced emotional engagement. The experience of being a passive observer also caused some students to not get emotionally involved. These students reported that they would have been more frightened if they had felt like they were actively participating in the video scenario. Moreover, some students were overwhelmed by the new technology. They mentioned that they could not focus on the content of the video due to feeling overwhelmed by the immersive virtual reality experience.

Further, the term “safety” was mentioned by several students. They expressed that they felt safe knowing that their presence was of no relevance in the scenario. This helped them maintain focus on the course of action in the portrayed scenario.

I felt like I got more of an understanding (...), let's say I think he (the father in the video scenario) overreacted, but then I sort of understood why he was so frustrated. Early on, I felt sympathy or compassion for his frustration of being a parent and not feeling that you are being understood. (...) when I felt like I was standing there, I got a totally different understanding than if I had only seen the letter (given to the father by the kindergarten) and read about it. I understood why he reacted in that way.

Student 1, Interview 5

### Debrief Sessions Enhance Comprehensive Learning

Overall, the students considered the subsequent reflection that occurred during the group debrief sessions a vital part of the virtual reality simulation. Watching the video as a separate part of their learning was useful, but the students regarded the subsequent reflection with fellow students during the group debrief sessions as the central component of their learning. The discussions facilitated explorations of the different perspectives of the actors involved in the scenario observed in the 360° video as well as about how the service provider acted as a professional in the situation. The students reported that they managed to expand their own individual perspectives through discussions with other students and that, together, they were able to analyze the portrayed situation deeper:

The students highlighted that their experiences and opinions (observations, interpretations, and emotional reactions) were different from each other, as they were individual learning experiences. This became apparent during the group debrief sessions. They were intrigued by their fellow students’ opinions and perspectives, which led to interesting and professional reflections and learning. While watching the video, the students had subjective interpretations, which were shared and reflected upon as well as challenged during the discussions with their fellow students. Being able to explore several perspectives was claimed to increase some of the students’ professional understanding.

It was very good to be able to properly break down what had happened and how you interpreted it and hear how the others had interpreted it. And then talk through all their perspectives. I felt that after that session, I had a much broader perspective on the whole situation.

Student 9, Interview 1

Further, students experienced greater motivation to participate in the group debrief discussion than they usually did to participate in an individual written assignment. They expressed that a virtual reality simulation conducted in groups as a learning activity was more engaging than traditional teacher-led lessons. They expressed that discussing the scenario in small groups was more facilitative for discussing their different opinions and observations than having to raise their hand in a conventional lecture in front of many students. The group discussion promoted the sharing of thoughts, experiences, and feelings:

You can’t say anything wrong in a discussion like this. There is nothing wrong when it comes to your own experiences or emotions.

Student 2, Interview 4

Notably, the facilitator was considered vital for keeping the discussion going, which was reflected by how one of the students indicated that the presence of the facilitator was important to help them maintain a professional focus during the discussion. Another student stated that the facilitator kept them on point and did not let them stray from the topic.

Our facilitators didn’t contribute with their own perspective. However, they asked open questions for us to find our own perspectives and suggestions for solutions.

Student 4, Interview 4

Several students reported how reflections on the role of the professional helper were useful to better prepare them to face a similar situation in real life. Several students reported that the combination of the simulation and the group discussions provided them with important experience and that they now felt better equipped to handle a similar situation.

I think it helps me to be more reflective. I don't often think through what I do and don't do. So, for me, it's good to become aware of what I do.

Student 1, Interview 2

## Discussion

### Principal Findings

This study provides insight into the students’ perspectives regarding the “Solstien 3” virtual reality simulation learning activity, which was piloted with students from the 4 undergraduate programs of nursing, occupational therapy, social education, and social work. Our results indicate that students believe that 360° videos provide observations that enhance their individual learning, that such videos activate emotional learning, and that group debrief sessions enhance comprehensive learning. We discuss these results in relation to earlier research on virtual reality simulation as well as to Illeris’ theory of learning and the dimensions of cognition, emotion, and sociality.

### Comparison With Prior Work

#### Sensory and Emotional Reactions for Cognitive Processing

Our results show that the students’ observations enabled by the virtual reality simulation may facilitate individual reflection on the portrayed situation. Through sensory experiences and impressions, the students were required to individually reflect upon clinically relevant cases for which professional skills are needed. The observer role may stimulate cognitive processing. According to Illeris [17], the cognitive dimension in the learning process refers to enhancing one’s knowledge and understanding through thinking, reasoning, and problem solving. Virtual reality simulation enables learners to expand their knowledge and understanding by presenting a case in which there is a need for them to use cognitive processing and reflection to explore a given situation. The scenario presented in the 360° video represents a situation in which the students were challenged to reflect, critically evaluate, reason, and problem solve. These processes provide them with an understanding of the purpose behind the learning activity, which Illeris [17] claims is central for learning. Illeris' theory suggests that learning involves a combination of cognitive, emotional, and social aspects and that meaningful learning often arises from experiences that challenge and engage the learner. In the context of training, repeated training could potentially be seen as a form of spaced repetition, in which learners revisit material over time to reinforce their understanding. This aligns with the idea that reflection and repeated exposure contribute to deeper learning [18]. As for volume training, it could relate to the idea that extended practice and exposure to a skill or knowledge area can lead to expertise through ongoing refinement and improvement. Illeris' theory underscores the significance of practice and application in the learning process.

Our results align with earlier research that found virtual reality simulation to be engaging and induce the process of clinical reasoning for students [10]. Higher education programs in health care and social work may benefit from using virtual reality simulation in learning situations to ensure that students develop essential nontechnical skills, such as communication skills, ethical reflection, and problem solving [1]. Our results reveal how students used the virtual reality simulation to observe and reflect, which in turn, allowed the students to gain a deeper understanding compared with more traditional learning activities such as written assignments.

Although virtual reality simulation offers advantages such as creating sensory reactions that are beneficial for learning, our results also show that some students experienced disturbances, such as noises caused by other students, when watching the 360° video on the virtual reality headset. This was reported to reduce their sense of realism and immersion, indicating that the way the virtual reality simulation was organized was not optimal for all students. This result highlights the importance of optimizing the implementation of such activities in the educational setting to avoid hindrances that may interrupt or diminish the learning outcome for the students, which has also been pointed out in earlier research [27]. Such distractions may negatively impact the effectiveness of virtual reality simulation. To prevent noise-related distractions, we suggest that students wear additional audio headsets when watching 360° videos in groups of several students to remove some of the disturbances. Additionally, limiting the number of students in each room is also an effective way to reduce noise.

Our results show that the 360° video experience was an emotionally activating experience. According to Illeris [18], emotions play a central role in the learning process. In our immersive virtual reality–based learning activity, the students were exposed to emotionally activating stimuli that engaged them. Furthermore, the observer position also made them feel safe, which can positively influence learning. Illeris [17] stated that emotional impulses are stored in the students’ mental schema and that these experiences can be used to build upon their existing knowledge and experiences. When learners use and build on their previous experiences, they may feel competent, which can enhance their motivation regarding the learning process [18,28]. Students who have a positive emotional experience in the learning situation, such as a feeling of engagement or safety, are more likely to retain and use the information they have learned [17].

Our results build upon previous research that has shown that immersive 360° environments can positively affect students’ emotional response to the learning climate, leading to improved attention, engagement, and motivation to learn [1].

Our results also demonstrate that some students found virtual reality technology to be overwhelming. In addition, some students mentioned that they had been provided with too much information about the video before they watched it. This reduced the element of surprise, leading to a decrease in their emotional activation. It seems important to provide proper training on the use of virtual reality equipment to ensure that learners can maintain focus on the simulation and not be distracted nor overwhelmed by the technology, as this has also been reported as a success factor for the implementation of such activities in previous research [7]. Therefore, we recommend that faculty members give students sufficient technological training and practical information prior to virtual reality simulation. Facilitators may keep the information regarding the scenario to a minimum to not lose the element of surprise before the simulation occurs [18].

#### The Debrief After the 360° Video Creates an Environment for Comprehensive Learning

A recent systematic review pointed out that additional research is needed to determine which debriefing methods are most effective for virtual simulations [29]. Our results show that face-to-face debriefing in groups was seen as essential in the present virtual reality simulation, and this is valuable for the evidence base concerning VR simulation. During these sessions, students’ perspectives were broadened as they exchanged thoughts, feelings, and observations and gained a better understanding of the portrayed situation. Although all the students watched the same video, they expressed different experiences and emotional responses to the events shown. This led them to be intrigued about why their peers perceived the scenario differently. The perception of a scenario can vary based on a student’s experiences, knowledge, and ability to observe. As Illeris [18] claimed, group learning is effective because students learn from each other’s different perspectives. Group activities also foster a sense of togetherness and motivation to learn and may enhance social skills and empathy [30]. For effective group learning, it is crucial for group members to be willing to cooperate and learn from one another.

Furthermore, our results indicate that the students were more motivated to learn through the group debrief sessions after watching the 360° video compared with other, more conventional learning activities, such as individual written assignments. This highlights the potential benefits of collaborative learning, particularly in settings where students engage with each other and exchange ideas. Collaborative learning refers to situations in which students are taught in groups but not necessarily to perform a team task. The idea is that there are features of group interaction that benefit the learning process (eg, the opportunity for vicarious learning or interaction with peers) [15]. Additionally, our results suggest that the “Solstien 3” virtual reality simulation learning activity provides a safe and controlled environment for students to achieve learning.

According to the ”Healthcare Simulations Standards of Best Practice,“ the facilitator’s role is to help students develop skills and conduct critical thinking and problem solving both during and after a simulation-based activity, through debriefing [31,32]. A facilitator can help establish a supportive framework for student interactions, which can increase motivation and positively affect learning [18]. Our results indicate that there is a clear necessity for and added value of a facilitated debriefing in the virtual reality simulation. Our results build upon this evidence and suggest that teacher-facilitated group discussions optimize learning from virtual reality simulation by creating a safe space for students to express their thoughts and feelings. This indicates that the group discussions as part of virtual reality simulation stimulates relationships, communication, and group dynamics, explained as central to learning by Illeris [18] as part of the *social dimension of learning*.

In summary, our study indicates that virtual reality simulation made it possible for students to observe, be emotionally activated by, and thereafter reflect on a portrayed situation. The visual impressions were important for individual reflections. Furthermore, the facilitated group debrief sessions were experienced as an essential part of learning, because they promoted collaborative learning. All these aspects allowed students to gain a deeper understanding of the scenario presented in the 360° video as compared with traditional lectures. However, due to some experiences with disturbances, we recommend that, when large student groups watch 360° videos in head-mounted displays, it is of value to wear additional audio headsets to prevent noise-related distractions. Additionally, limiting the number of students in each group can be of value. Moreover, for the best possible learning experience, sufficient technological training and practical information prior to virtual reality simulation are vital.

In future research, larger usability studies on students’ experiences with virtual reality simulation are recommended. Moreover, the long-term impact of virtual reality simulation on students’ learning should be explored. More in-depth studies, including both qualitative and quantitative studies, could be conducted to analyze how virtual reality simulation promotes the learning of clinical skills, as well the relationship between students’ approaches to learning and their experience with virtual reality in simulation learning activities. It will also be of value to compare virtual reality simulation with more standard simulation methodologies when it comes to learning outcomes and experiences.

### Limitations

Generalizations from this qualitative study are not possible nor intended. In this study, 6 focus groups were conducted by different moderators, and each consisted of 1 interviewer and 1 facilitator. All the focus group followed the same semistructured guide, although the number of students present in each group varied due to practical reasons, with 3 to 7 students in each group. In addition, 7 of the students who volunteered for the simulation did not accept the invitation to participate in the interviews. The focus groups were conducted after the learning activity, in which the moderators served as facilitators. Relationships between students and moderators were therefore established ahead of the focus groups, which may have affected the students' responses. Moreover, students who have more positive attitudes toward using VR technology in education may tend to volunteer more often and thus be overrepresented in the sample. This may have influenced the focus group discussions and therefore our results. We were aware of this challenge during the interviews, and the informants were therefore encouraged to reflect critically and describe challenges and suggestions for improvement or changes.

### Conclusions

In conclusion, students had a positive experience with the virtual reality simulation in the context of the “Solstien 3” learning activity. From the students’ points of view, virtual reality simulation is a valuable contribution to health care and social work education, as it enables observations for individual learning and activates emotional learning. Facilitated group debrief sessions were highlighted as being a central part of the learning experience that allow students to explore different perspectives and expand their own understanding, which supports comprehensive learning.

Our main result indicates that the use of 360° videos in combination with group discussions as a virtual reality simulation learning activity appears to be promising for enhancing the professional learning of health care and social work students. The 3 mentioned dimensions from Illeris’ [17] theory seem to stimulate increased learning. Our results add to the body of knowledge on virtual reality simulation as an important tool for improving learning outcomes and competence [3,12]. This may, in turn, improve the quality of health care and social services [4].

## Appendix

Multimedia Appendix 1 Interview guide.

Multimedia Appendix 2 Consolidated Criteria for Reporting Qualitative Studies (COREQ) 32-item checklist.

Multimedia Appendix 3 Coding tree.

## Abbreviations

COREQ: Consolidated Criteria for Reporting Qualitative Studies
PEARLS: Promoting Excellence and Reflective Learning in Simulation

## Data Availability

Due to ethical concerns, access to the data has been restricted, as emphasized in the participants’ informed consent form. The data consist of transcriptions in the Norwegian language.
