# Supplementary material for: Health Care and Social Work Students’ Experiences With a Virtual Reality Simulation Learning Activity: Qualitative Study
Source: JMIR Med Educ. 2023 Sep 20;9:e49372. doi: 10.2196/49372 (PMC10551784; doi:10.2196/49372)
Supplement: Multimedia Appendix 1 [file mededu_v9i1e49372_app1.docx]

Interview guide for focus group interviews with students in occupational therapy, social work, nursing, and social education regarding the testing of "Solstien 3."

1. Participation in testing of VR goggles and learning activities

You have watched films through VR goggles and participated in learning activities – could you tell us a bit about how it was?

• What engaged you – what caught your interest?

o If you were engaged – please elaborate.

o If you didn't like this setup very much – please explain why.

2. Experiences with the technical and sensory aspects of the VR goggles

Could you tell us how the technical aspects worked? How did the equipment function?

• Did you experience any discomfort, dizziness or other bodily reactions while using the VR goggles?

3. How did you experience the events in the VR films?

• When you watched the VR films – did you feel like you were part of the situations?

o How were the sensory impressions?

o Can you share a specific experience you had here?

o How does VR create shared experiences?

o How did you feel that VR enables training in challenging situations?

o Can you describe what it was like to be in the room – in the actual film?

o How realistic did it feel? What did you feel during the experience?

o How do you feel that the scenarios generate engagement, curiosity, and emotions? (motivation to learn more?)

• Which emotional expressions made a particularly strong impression on you?

o Were there any reactions you received that surprised you?

4. Can you tell us how you experienced the associated learning activities?

• How did you experience the associated tasks?

o Is there anything you remember particularly well that engaged you?

- Can you elaborate on your experiences?

- If you weren't engaged, could you tell us a bit about that?

• How did you experience reflecting on shared experiences afterward?

o What did you learn from the activities afterwards? Can you provide examples?

• What opportunities does this setup offer for professional growth?

- How can you use what you learned in practice?

- What theories/knowledge did you apply in the tasks?

• How do the associated tasks create engagement, curiosity, and emotions? (motivation for learning?)

• How did you experience the collaboration with the teacher in this setting?

5. Learning outcomes: Interprofessional collaboration, ethical challenges, and experiences in relationships and communication

5.1 Ethical challenges and dilemmas

When you work as social educators, nurses, occupational therapists, and social workers, you will encounter various ethical dilemmas where you wonder and have to assess what is right to do, meaning what is best in the situations. When you watched these films (and completed the tasks), was there anything that you thought was challenging or difficult? Can you tell us a bit about that?

• Was there anything that happened where you thought, "I'm worried about encountering this in practice" or "Here, I don't know what I would have said or done"? Please share.

• Based on what you saw, did you get ideas about what you could do or can do if you encounter such situations? (or do you hope it doesn't happen at all).

o Did you learn anything from the film related to ethical dilemmas/reflections? Does this affect your perception of competence for practice?

• Was there anything you thought the character in the film should have solved differently? Or did you think, "I would have done this differently"? Please tell us.

• Did you identify any ethical dilemmas in the film? Please share.

• Based on what you experienced here: What kind of knowledge do you need to uphold your values as healthcare professionals and/or social workers?

5.2 Interprofessional collaboration

When you work as social educators, nurses, occupational therapists, and social workers, you will have to collaborate with each other as professionals. When you worked on the tasks together as a group, how did this collaboration go? Was there anything you thought went well? Was there anything that was challenging, difficult? Can you tell us a bit about that?

• How do you think that the VR films and associated setup contribute to achieving competence in interprofessional collaboration?

• How can you address different value conflicts interprofessionally?

• How did you experience the interactions between the people involved in the film and their surroundings?

• Do you feel that what you experienced by watching the films and working with the different learning activities has given you competence in collaboration that can be used in practice? If not, what would you have needed for that to happen?

• Based on what you have experienced now, did it provide any idea of how to develop action plans in challenging situations?

• In what way can the experience you've gained now contribute to better interprofessional collaboration?

• Based on what you experienced here: What kind of knowledge do you need to uphold your values as healthcare professionals and/or social workers in interprofessional collaboration?

5.3 Experiences in relationships and communication

When you work as social educators, nurses, occupational therapists, and social workers, you will work with relationships and communication with colleagues, patients/users, family members, and more. When you watched these films (and completed the tasks), was there anything you thought went well? Was there anything that was challenging, difficult? Can you tell us a bit about that?

• Did you learn anything in this situation that you can use in practice going forward?

• What kind of knowledge is needed to uphold the patient's right to user involvement?

• What does it mean to you to be professional in relation to patients/users and their families?

• Tell us about an episode during the work with "Solstien 3" where you learned something specific related to relationship and communication skills.

o What could have been done differently?

o By you as students or in the setup?

• Based on what you experienced here: What kind of knowledge do you need to establish good relationships and communicate effectively in practice?

Conclusion:

What advice would you give to improve or make the use of Solstien 3 more useful?
