# Supplementary material for: Health Care and Social Work Students’ Experiences With a Virtual Reality Simulation Learning Activity: Qualitative Study
Source: JMIR Med Educ. 2023 Sep 20;9:e49372. doi: 10.2196/49372 (PMC10551784; doi:10.2196/49372)
Supplement: Multimedia Appendix 2 [file mededu_v9i1e49372_app2.docx]

Multimedia appendix II: Consolidated criteria for reporting qualitative studies (COREQ): 32-item checklist [26]

| **No** | **Item** | | **Question** | | | **Answer** | | | | **Reported in** |
| --- | --- | --- | --- | --- | --- | --- | --- | --- | --- | --- |
| **Domain 1: Research team and reflexivity** | | | | | | | | | |  |
| **Personal characteristics** | | | | | | | | | |  |
| 1 | Interviewer/facilitator | | Which author/s conducted the interview or focus group? | | | Nikolina Helle, Miriam Dubland Vikman and Silje Stangeland Lie + colleagues Ole Sønnik Dydland Larsen, Renate Alvestad, Hans Martin Kunnikoff, Elise Hauge, Astrid Flacke, Geir Tarje Fugleberg Bruaset | | | | Data collection, page 4, and acknowledgement page 10 |
| 2 | Credentials | | What were the researcher’s credentials? | | | NH & MDV : MSc  Co-authors 3 TDM &4 SSL: PhD | | | | Reported in author metadata |
| 3 | Occupation | | What was their occupation at the time of the study? | | | First authors: Assistant professors  Co-authors: Associate professor (SSL) and professor (TDM) | | | | Reported in author metadata |
| 4 | Gender | | Was the researcher male or female? | | | Females | | | | Considered not relevant, reported only by name |
| 5 | Experience and training | | What experience or training did the researcher have? | | | First authors: Novice  Co-authors: Experienced | | | | Page 4 and author metadata |
| **Relationship with participants** | | | | | | | | | |  |
| 6 | Relationship established | | Was a relationship established prior to study commencement? | | | Yes | | | | Page 10 (limitations) |
| 7 | Participant knowledge of the interviewer | | What did the participants know about the researcher? e.g. personal goals, reasons for doing the research | | | They were informed about the reasons for doing the research both verbally and by the written information | | | | Page 4 |
| 8 | Interviewer characteristics | | What characteristics were reported about the interviewer/facilitator? e.g. Bias, assumptions, reasons and interests in the research topic | | | They were informed about the reasons for doing the research both verbally and by the written information, and in the focus group all presented themselves | | | | Page 4 |
| **Domain 2: Study design** | | | | | | | | |  | |
| **Theoretical framework** | | | | | | | | |  | |
| 9 | Methodological orientation and Theory | | What methodological orientation was stated to underpin the study? | | Qualitative design (thematic analysis)  Theory is described in introduction | | | | Page 5-6, data-analysis  Page 2-3 | |
| **Participant selection** | | | | | | | | |  | |
| 10 | Sampling | | How were participants selected? | | | | Students who voluntarily participated in the pilot of “Solstien 3” were asked to participate in the focus groups. 28 out of 35 participated. | | Page 4-5 | |
| 11 | Method of approach | | How were participants approached? | | | | Asked verbally an in writing ahead of the pilot of “Solstien 3” | | Page 4 | |
| 12 | Sample size | | How many participants were in the study? | | | | 28 | | Page 4 | |
| 13 | Non-participation | | How many people refused to participate or dropped out? Reasons? | | | | 7 did not want to participate in focus group | | Page 4 | |
| **Setting** | | | | | | | |  | | |
| 14 | | Setting of data-collection | | Where was the data collected? | | | Six qualitative focus group interviews at the university | Page 4 | | |
| 15 | | Presence of non-participants | | Was anyone else present besides the participants and researchers? | | | No | n/a | | |
| 16 | | Description of sample | | What are the important characteristics of the sample? | | | Students | Page 4 | | |
| **Data collection** | | | | | | | |  | | |
| 17 | | Interview guide | | Were questions, prompts, guides provided by the authors? Was it pilot tested? | | | We had a semistructured interview guide | Page 4 | | |
| 18 | | Repeat interviews | | Were repeat interviews carried out? If yes, how many? | | | No | n/a | | |
| 19 | | Audio/visual recording | | Did the research use audio or visual recording to collect the data? | | | Yes, audio recordings | Page 4 | | |
| 20 | | Field notes | | Were field notes made during and/or after the interview or focus group? | | | Yes | n/a | | |
| 21 | | Duration | | What was the duration of the interviews or focus group? | | | Reported | Page 4 | | |
| 22 | | Data saturation | | Was data saturation discussed? | | | No, as we interviewed the entire “sample” that participated in the VR simulation, data saturation is not relevant | n/a | | |
| 23 | | Transcripts returned | | Were transcripts returned to participants for comment and/or correction? | | | No | n/a | | |

| **Domain 3: analysis and findings** | | | |  |
| --- | --- | --- | --- | --- |
| **Data analysis** | | | |  |
| 24 | Number of data coders | How many data coders coded the data? | All authors (n=4) | Page 5-6 |
| 25 | Description of the coding tree | Did authors provide a description of the coding tree? | Verbal description in methods section | Not presented in article, but coding tree can be retrieved from authors |
| 26 | Derivation of themes | Were themes identified in advance or derived from the data? | Data-driven | Page 5-6 |
| 27 | Software | What software, if applicable, was used to manage the data? | Word | n/a |
| 28 | Participant checking | Did participants provide feedback on the findings? | No |  |
| 29 | Quotations presented | Were participant quotations presented to illustrate the themes/findings? Was each quotation identified? e.g. participant number | yes | Results section page 6-8 |
| 30 | Data and findings consistent | Was there consistency between the data presented and the findings? | Yes | Illustrated by presenting quotations |
| 31 | Clarity of major themes | Were major themes clearly presented in the findings? | Yes | Themes and codes are presented in findings, page 6-8 |
| 32 | Clarity of minor themes | Is there a description of diverse cases or discussion of minor themes? | Yes |  |
