# Supplementary figures and images for: Health Care and Social Work Students’ Experiences With a Virtual Reality Simulation Learning Activity: Qualitative Study
Source: JMIR Med Educ. 2023 Sep 20;9:e49372. doi: 10.2196/49372 (PMC10551784; doi:10.2196/49372)

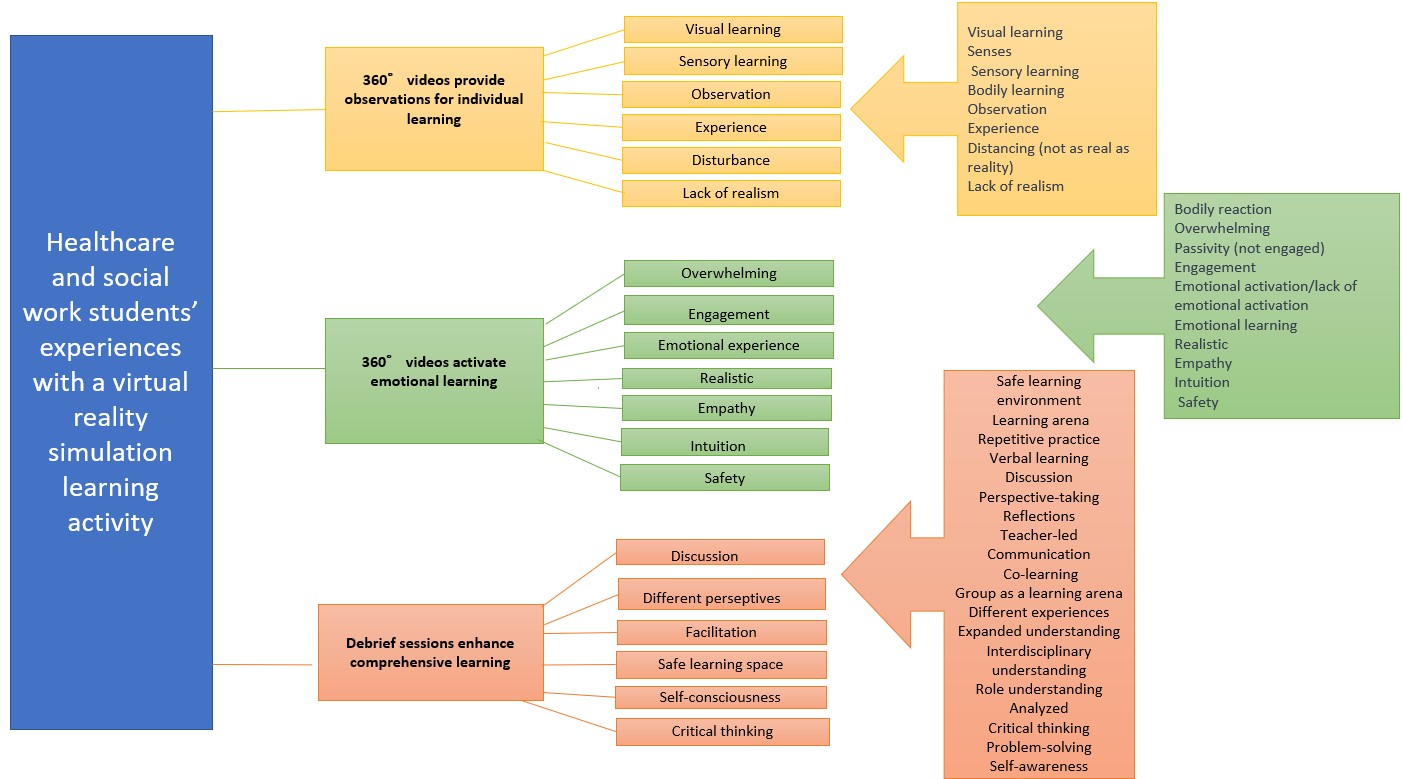

Supplement: Multimedia Appendix 3 [file mededu_v9i1e49372_app3.png]
